# Supplementary material for: Development of a new flippase-dependent mouse model for red fluorescence-based isolation of KRASG12D oncogene-expressing tumor cells
Source: Transgenic Res. 2025 Jan 9;34(1):9. doi: 10.1007/s11248-024-00429-2 (PMC11717838; doi:10.1007/s11248-024-00429-2)
Supplement: Supplementary file 1 — Supplementary file1 (DOCX 17 KB) [file 11248_2024_429_MOESM1_ESM.docx]

**Supplementary information**

**Supplementary video**

Video 1: Fluorescence of 3-dimensional Z-scans showing whole-mount staining of E-cadherin (green) and RFP/tdTomato (red) in the lung sample (thickness ~50 µm) of *Kras^wt/RR^* mice 24 weeks after infection with Ad-Flpo. Video 2: *Kras^wt/wt^* tissue shows only E-cadherin positivity and no RFP/tdTomato signal. The cell nuclei were counterstained with DAPI (blue). The yellow arrows show the direction of the Z-scan image. Scale bar: 50 µm.

**Supplementary Figure S1**

Analysis of expression levels of *Kras^wt^* and *Kras^RR^* mRNA in small intestinal organoids derived from *Kras^wt/Flp-ready^* mice. Two organoid cultures (#1 and #2) derived independently were tested prior (Ad-Flpe -) and after (Ad-Flpe +) Ad-Flpe infection. After the infection, organoids were grown in the presence of the EGFR receptor inhibitor gefitinib for two passages and analyzed by RT-PCR. A PCR fragment encoding the N-terminal fragment of KRAS was amplified by primers comprising exons 1 to 4 present in both *Kras^wt^* and *Kras^RR^* mRNA. Recombined organoids express comparable levels of *Kras^wt^* and *Kras^RR^* transcripts (325 pb and 445 bp, respectively). In contrast, non-recombined *Kras^wt/Flp-ready^* organoids express only the wt *Kras* transcript. M, molecular weight marker.
